# Very Long‐Term Outcomes of Laparoscopic Adjustable Gastric Banding in a Japanese Cohort: A Single‐Center 15‐Year Follow‐Up Study

**DOI:** 10.1002/ags3.70244

**Published:** 2026-07-08

**Authors:** Yuichi Endo, Hiroomi Takayama, Wataru Miyoshino, Shun Nakamura, Yuiko Nagasawa, Masahiro Kawamura, Yoko Kawano, Takashi Masuda, Teijiro Hirashita, Yoshinori Ozeki, Takayuki Masaki, Hirotaka Shibata, Masafumi Inomata

**Affiliations:** ^1^ Department of Gastroenterological and Pediatric Surgery, Faculty of Medicine Oita University Yufu Oita Japan; ^2^ Obesity and Diabetes Center for Advanced Medicine, Faculty of Medicine Oita University Yufu Oita Japan; ^3^ Department of Endocrinology, Metabolism, Rheumatology and Nephrology, Faculty of Medicine Oita University Yufu Oita Japan

**Keywords:** Asian population, bariatric surgery, laparoscopic adjustable gastric banding, long‐term outcomes, metabolic comorbidity, reoperation

## Abstract

**Background:**

The long‐term clinical role of laparoscopic adjustable gastric banding (LAGB) remains uncertain in the contemporary era of metabolic surgery, and very long‐term outcome data from Asian populations are limited. This study evaluated very long‐term outcomes of LAGB in a Japanese cohort, focusing on weight change, metabolic comorbidities, and reoperations.

**Methods:**

We retrospectively reviewed consecutive patients who underwent LAGB between 2005 and 2010 at a single institution. Changes in body weight, metabolic comorbidities, and reoperation were assessed for up to 15 years. Weight outcomes were evaluated using percent total weight loss (%TWL) and percent excess weight loss (%EWL).

**Results:**

A total of 28 patients were included. Mean %EWL was 49% at 1 year, 62% at 5 years, 64% at 10 years, and 59% at 15 years, indicating sustained long‐term weight reduction despite partial weight regain. Early improvements in metabolic comorbidities were observed; however, recurrence of diabetes mellitus, hypertension, and dyslipidemia increased after 5–10 years, whereas hepatic comorbidities remained relatively stable. Reoperation was required in 7 of 28 patients (25%), mainly because of device‐related complications or insufficient weight loss.

**Conclusion:**

This study provides very long‐term outcome data of LAGB in an Asian cohort. Although LAGB achieved sustained weight reduction in selected patients, metabolic improvements showed limited long‐term durability and a substantial proportion of patients required reoperation. These findings highlight the limitations of a purely restrictive and device‐dependent bariatric procedure in the contemporary era of metabolic surgery.

## Graphical abstract

Long‐term outcomes of laparoscopic adjustable gastric banding (LAGB) in Japan were retrospectively evaluated in 28 patients with up to 15 years of follow‐up. LAGB achieved sustained long‐term weight reduction in selected patients; however, recurrence of metabolic comorbidities increased during extended follow‐up. Reoperation was required in 25% of patients, mainly because of device‐related complications or insufficient weight loss. These findings highlight the limited long‐term metabolic durability and device‐dependent limitations of LAGB in the contemporary era of metabolic surgery.

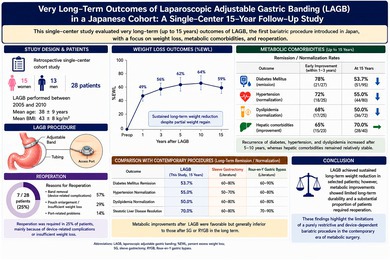

## 1 Introduction

Obesity is a chronic, progressive disease associated with substantial morbidity and mortality, largely driven by obesity‐related metabolic disorders such as type 2 diabetes mellitus, hypertension, dyslipidemia, and steatotic liver disease. Conventional medical therapies often fail to achieve sustained long‐term weight reduction in patients with severe obesity. Bariatric and metabolic surgery has therefore become an established and effective treatment for severe obesity worldwide, providing durable weight reduction and improvement of metabolic comorbidities. However, bariatric surgery has historically been less prevalent in Asian populations than in Western countries, and very long‐term outcome data from Asian cohorts remain limited. Over the past two decades, the concept of metabolic surgery has further evolved, emphasizing its role not only in weight reduction but also in the improvement and potential remission of metabolic disease [1, 2, 3].

LAGB was widely adopted as a bariatric procedure because of its technical simplicity, reversibility, and favorable perioperative safety profile. As a purely restrictive operation, LAGB limits food intake by placing an adjustable band around the proximal stomach, allowing postoperative modification of restriction. During the early era of bariatric surgery, LAGB became one of the most commonly performed procedures worldwide and was considered an attractive option because it avoided gastrointestinal resection [4, 5].

Despite its widespread use in the past, the long‐term effectiveness of LAGB remains a matter of debate. Although sustained weight reduction has been reported in selected patients, concerns have emerged regarding the durability of metabolic improvement and the need for reoperation during extended follow‐up [4, 6, 7]. In parallel with accumulating evidence favoring procedures with greater metabolic effects [2, 3], the use of LAGB has declined substantially worldwide over the past decade, being largely replaced by sleeve gastrectomy and gastric bypass procedures [8]. However, data describing very long‐term outcomes of LAGB, particularly in Asian populations, remain limited [9].

The aim of this study was to evaluate the very long‐term outcomes of LAGB in a Japanese cohort. Specifically, we assessed longitudinal changes in body weight, obesity‐related comorbidities, and reoperations over extended follow‐up.

## 2 Methods

### 2.1 Study Design and Patients

This was a single‐center retrospective study conducted at our institution.

Between 2005 and 2010, 30 patients underwent LAGB. Two patients were excluded because they were foreign nationals who returned to their home country soon after surgery, leaving 28 patients for analysis.

A total of 28 consecutive patients who underwent LAGB between 2005 and 2010 were retrospectively enrolled in this study. Follow‐up data were collected through December 2025, allowing a maximum postoperative follow‐up duration of 15 years. Baseline patient characteristics, including age, sex, preoperative body mass index (BMI), and obesity‐related comorbidities, are summarized in Table 1. All baseline variables were obtained from medical records at the time of surgery.

**TABLE 1:** Baseline characteristics of patients who underwent laparoscopic adjustable gastric banding.

Number of patients	28
Age	38 ± 9
Gender (F/M)	15/13
Preoperative body weight (kg)	117 ± 6
Preoperative BMI (kg/m2)	43.4 ± 8
Comorbidities	
Diabetes mellitus	10 (28%)
Hypertension	16 (57%)
Dyslipidemia	17 (61%)
Steatotic liver disease	10 (36%)
Liver dysfunction	21 (75%)

*Note:* Data are presented as mean ± standard deviation unless otherwise indicated.

Abbreviation: BMI, body mass index.

The study was conducted in accordance with the principles of the Declaration of Helsinki and was approved by the institutional review board. Study information was disclosed on the institutional website, and patients were provided with the opportunity to opt out of participation in accordance with institutional ethical guidelines. At the time of introduction, the LAP‐BAND system had not yet been approved for routine clinical use in Japan. Therefore, the procedure was performed under institutional ethical committee approval with detailed informed consent obtained from all patients. The ethical review included perioperative management and management of postoperative complications and revisional procedures. Treatment‐related costs were explained to patients during the informed consent process in accordance with institutional regulations at that time. The requirement for written informed consent was waived due to the retrospective nature of the study.

### 2.2 Surgical Procedure

LAGB was performed using a standardized technique as previously described [10]. A commercially available adjustable gastric band system (LAP‐BAND system: Allergan Medical, Santa Barbara, USA) was placed around the proximal stomach to create a small gastric pouch. The band was connected to a subcutaneous access port, allowing postoperative adjustment.

Postoperative band adjustments were performed as clinically indicated based on weight loss progress, symptoms, and radiologic or clinical findings.

### 2.3 Outcome Measures

#### 2.3.1 Weight Loss Outcomes

Weight loss outcomes were evaluated using percentage total weight loss (%TWL) and percentage excess weight loss (%EWL).

*%TWL* was calculated as:

%TWL = [(preoperative weight − postoperative weight) / preoperative weight] × 100.

%EWL was calculated as:

%EWL = [(preoperative weight − postoperative weight)/(preoperative weight − ideal body weight)] × 100.

Ideal body weight was defined as the body weight corresponding to a body mass index (BMI) of 25 kg/m^2^.

Weight loss outcomes and follow‐up rates at 1, 3, 5, 10, and 15 years after surgery are summarized in Table 2.

**TABLE 2:** Long‐term weight outcomes and follow‐up rates after laparoscopic adjustable gastric banding.

	1 year	3 years	5 years	10 years	15 years
% EWL	49	61	62	64	59
% TWL	20	25	24	26	25
TWL (kg)	24 ± 12	28 ± 11	28 ± 11	31 ± 20	31 ± 24
Follow up rate (%)	100	79	89	68	40

*Note:* Data are presented as mean ± standard deviation.

#### 2.3.2 Improvement of Obesity‐Related Comorbidities

**FIGURE 1:**
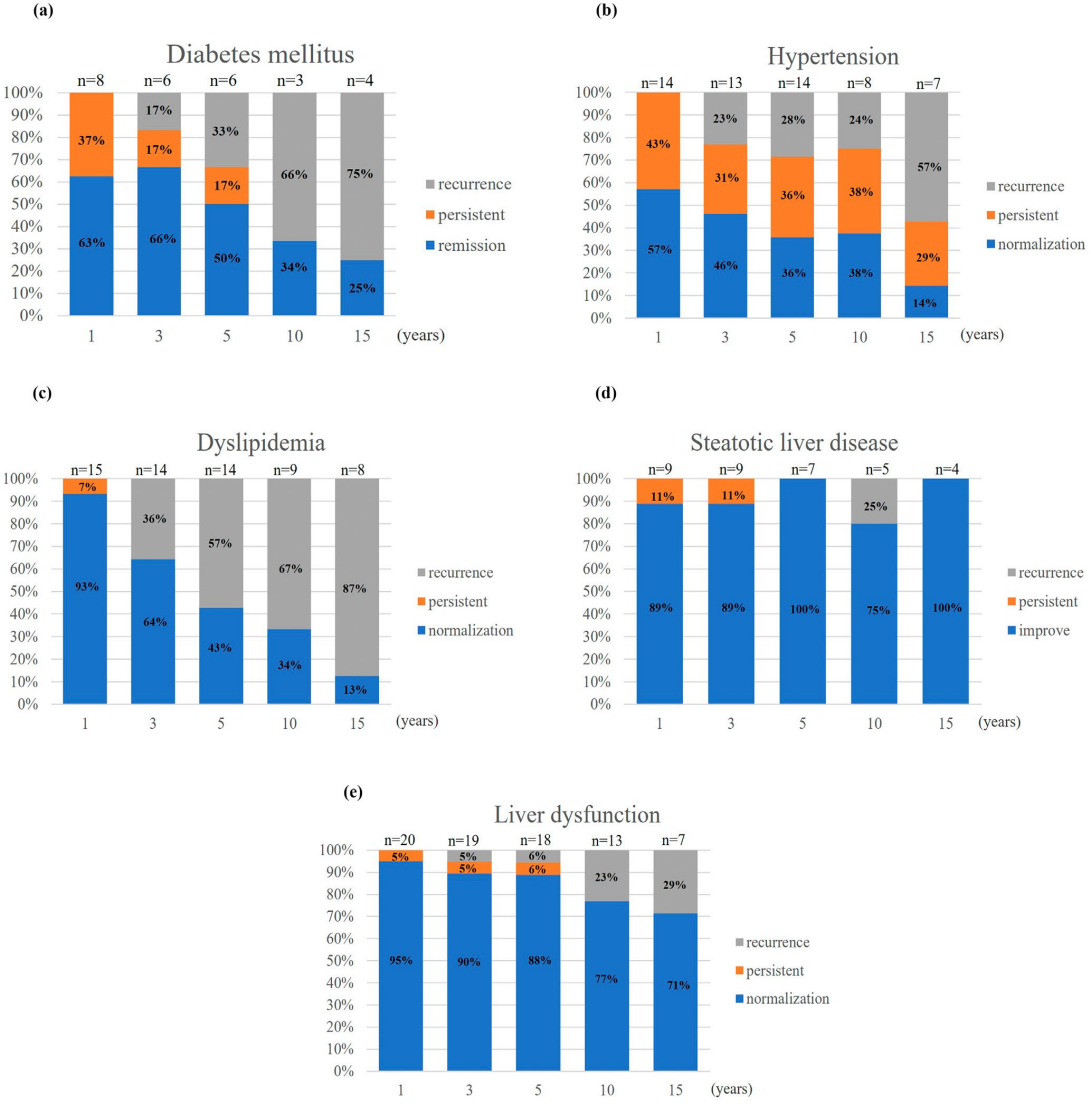
(a–e) Long‐term changes in obesity‐related comorbidities after laparoscopic adjustable gastric banding. Changes in the status of obesity‐related comorbidities during follow‐up are shown for (a) diabetes mellitus, (b) hypertension, (c) dyslipidemia, (d) steatotic liver disease, and (e) liver dysfunction. Remission, normalization, and improvement were defined according to the criteria described in the Methods section. Numbers above each bar indicate the number of evaluable patients at each postoperative time point. Persistent disease indicates lack of improvement during follow‐up according to the criteria defined for each outcome. New‐onset cases were excluded from the graphical presentation because of their small number.

Obesity‐related comorbidities evaluated in this study included diabetes mellitus, hypertension, dyslipidemia, steatotic liver disease, and liver dysfunction. Disease status was assessed longitudinally at 1, 3, 5, 10, and 15 years after surgery when data were available. Improvement rates are illustrated in Figure 1.

Definitions of obesity‐related comorbidities were based on previously reported criteria for bariatric outcomes as applied in our prior study [11].

#### 2.3.3 Diabetes Mellitus

Diabetes mellitus was defined based on medical records, laboratory data, and use of antidiabetic medications.

Remission of diabetes mellitus was defined as HbA1c < 6.5% without antidiabetic medication.

#### 2.3.4 Hypertension

Hypertension was defined by the use of antihypertensive medications.

Normalization of hypertension was defined as blood pressure < 140/90 mmHg without antihypertensive medication.

#### 2.3.5 Dyslipidemia

Dyslipidemia was defined by the use of lipid‐lowering medications or documented abnormal lipid profiles.

Normalization of dyslipidemia was defined as serum lipid levels within the normal range without lipid‐lowering medication.

#### 2.3.6 Steatotic Liver Disease

Steatotic liver disease was assessed using computed tomography findings. Hepatic steatosis was defined as a liver‐to‐spleen attenuation ratio < 0.9 on non‐contrast CT images [11]. Improvement was defined as an increase in the liver‐to‐spleen ratio compared with baseline, including normalization to a ratio ≥ 0.9.

#### 2.3.7 Liver Dysfunction

Liver dysfunction was defined as elevated serum liver enzyme levels. Normalization was defined as liver enzyme levels within the normal range.

#### 2.3.8 Reoperations

Reoperation was defined as any revisional or device‐related surgical procedure performed after the initial LAGB. The indication, type, and timing of reoperation were retrospectively reviewed from the medical records. Patients who underwent revisional sleeve gastrectomy after LAGB were excluded from subsequent analyses of weight loss and obesity‐related comorbidities from the time of revisional surgery onward.

### 2.4 Statistical Analysis

Continuous variables are presented as mean ± standard deviation, and categorical variables are presented as numbers and percentages. Statistical analyses were performed using JMP software (SAS Institute Inc., Cary, NC, USA). A two‐sided *p* value < 0.05 was considered statistically significant.

## 3 Results

### 3.1 Patient Characteristics and Follow‐Up

A total of 28 patients who underwent LAGB were included in this retrospective study.

Baseline patient characteristics are summarized in Table 1.

Long‐term follow‐up data were available for a substantial proportion of patients. Follow‐up rates were 100%, 79%, 89%, 68%, and 40% at 1, 3, 5, 10, and 15 years after surgery, respectively (Table 2). The transient increase in follow‐up rate at 5 years reflects the availability of outcome data from patients who returned for long‐term evaluation despite missing intermediate follow‐up visits. These follow‐up rates reflect the availability of clinical data at each time point rather than continuous observation, as some patients returned for intermittent long‐term evaluation despite missing intermediate visits.

### 3.2 Weight Loss Outcomes

Postoperative weight loss outcomes are summarized in Table 2. LAGB resulted in marked weight reduction during the early postoperative period, with the greatest weight loss observed within the first 1–3 years after surgery. Thereafter, weight loss gradually attenuated over time, and partial weight regain was observed during long‐term follow‐up in a subset of patients.

Mean %EWL was 49% at 1 year, 62% at 5 years, 64% at 10 years, and 59% at 15 years among patients with available data, indicating sustained long‐term weight reduction despite some degree of weight regain.

### 3.3 Improvement of Obesity‐Related Comorbidities

Longitudinal changes in obesity‐related comorbidities are illustrated in Figure 1. At 1 year after surgery, substantial improvement was observed in major metabolic comorbidities, including diabetes mellitus (Figure 1), hypertension (Figure 1b), and dyslipidemia (Figure 1c). Hepatic comorbidities, including steatotic liver disease (Figure 1d) and liver dysfunction (Figure 1e), also showed high improvement rates during the early postoperative period.

### 3.4 Long‐Term Decline in Metabolic Improvement

Despite favorable early outcomes, the improvement rates of metabolic comorbidities declined progressively during long‐term follow‐up. In patients with diabetes mellitus at baseline, the proportion of improved cases decreased after 3–5 years, with a marked reduction observed at 10 and 15 years (Figure 1). Similar trends were observed for hypertension and dyslipidemia (Figure 1b,c), indicating limited long‐term durability of metabolic improvement after LAGB.

In contrast, improvements in steatotic liver disease and liver dysfunction were relatively well maintained over time, with a substantial proportion of patients showing sustained improvement even during long‐term follow‐up (Figure 1d,e).

### 3.5 Recurrence and New Onset of Comorbidities

To further clarify long‐term disease trajectories, patients were evaluated according to their baseline disease status. Among patients with preexisting metabolic comorbidities, recurrence or worsening of disease became increasingly evident during long‐term follow‐up. In particular, the proportion of patients with recurrent diabetes mellitus, hypertension, and dyslipidemia increased substantially after 5 years postoperatively, accompanied by a decline in the proportion of remission, normalization, or improvement (Figure 1a–e). New‐onset metabolic comorbidities during long‐term follow‐up were uncommon and therefore are described in the text rather than graphically presented.

### 3.6 Reoperations

Reoperation was required in 7 of 28 patients (25%) during long‐term follow‐up. Details of the reoperations, including the time to reoperation, primary indication, and type of reoperative procedure for each case, are summarized in Table 3.

**TABLE 3:** Causes of reoperation and types of revisional procedures after laparoscopic adjustable gastric banding.

Case	Time to reoperation (months)	Primary reason for reoperation	Type of reoperation
1	43	Port‐related problem	Port replacement
2	45	Band slippage	Band removal + sleeve gastrectomy
3	72	Band slippage	Band removal + sleeve gastrectomy
4	121	Sufficient weight loss achieved	Band removal
5	126	Insufficient weight loss/weight regain	Band removal + sleeve gastrectomy
6	133	Insufficient weight loss/weight regain	Band removal + sleeve gastrectomy
7	200	Port‐related problem	Band removal

Reoperations were most commonly performed for band‐related complications, including band slippage and port‐related problems, as well as insufficient weight loss or weight regain. In one patient, the gastric band was electively removed after sufficient weight loss had been achieved.

### 3.7 Summary of Long‐Term Clinical Outcomes

Collectively, these results indicate that LAGB provides meaningful early weight loss and initial improvement of obesity‐related comorbidities; however, long‐term follow‐up revealed limited durability of metabolic improvement, particularly for diabetes mellitus and dyslipidemia. In addition, a substantial proportion of patients required reoperation during extended follow‐up, highlighting the long‐term limitations of LAGB as a purely restrictive and device‐dependent bariatric procedure.

## 4 Discussion

In this study, we evaluated the long‐term outcomes of LAGB with follow‐up extending up to 15 years in a Japanese cohort. Three principal findings emerged. First, LAGB achieved sustained long‐term weight reduction in a proportion of patients. Second, although obesity‐related comorbidities initially improved after surgery, recurrence and new onset of metabolic disease became increasingly evident during extended follow‐up. Third, a considerable proportion of patients required reoperation due to device‐related complications or insufficient weight loss. These findings provide insight into the long‐term clinical trajectory of LAGB as a purely restrictive bariatric procedure.

One notable observation was the durability of weight loss after LAGB. Many patients maintained clinically meaningful weight reduction for more than a decade, consistent with the continuous mechanical restriction provided by the adjustable band. Long‐term studies from Western countries have reported mean %EWL values of approximately 40%–50% after more than 10 years of follow‐up [4, 6]. In comparison, the mean %EWL of 59% at 15 years observed in the present cohort appears numerically favorable, although these findings should be interpreted cautiously given the small sample size and declining follow‐up rate at later time points. Nevertheless, these results indicate that durable weight reduction can be achieved in selected patients even during very long‐term follow‐up.

Despite these favorable weight outcomes, metabolic improvements were not consistently durable. Early postoperative improvements in diabetes mellitus, hypertension, and dyslipidemia were observed in many patients; however, recurrence and new onset of these conditions became increasingly common after 5–10 years. Similar discrepancies between weight loss and metabolic outcomes have been reported in observational studies comparing different bariatric procedures [12]. These findings suggest that sustained weight reduction alone may not ensure long‐term metabolic control.

This discrepancy likely reflects the restrictive mechanism of LAGB. Unlike metabolically active procedures such as Roux‐en‐Y gastric bypass (RYGB) and sleeve gastrectomy (SG), LAGB does not substantially alter gastrointestinal anatomy or enteroendocrine signaling. Previous studies have demonstrated that SG and RYGB induce significant changes in gut hormones including glucagon‐like peptide‐1, peptide YY, and ghrelin, which contribute to improved glucose metabolism and appetite regulation [13, 14, 15]. In contrast, LAGB primarily reduces caloric intake through mechanical restriction and therefore exerts relatively limited metabolic effects. Representative long‐term studies of contemporary metabolic procedures have demonstrated more durable metabolic remission than that observed after LAGB in the present study (Table 4). In particular, RYGB consistently showed superior long‐term remission of diabetes mellitus, hypertension, and dyslipidemia compared with restrictive procedures. Although LAGB demonstrated durable long‐term weight reduction in selected patients, the present findings suggest that the principal limitation of LAGB may be the limited metabolic effect of a purely restrictive procedure.

**TABLE 4:** Representative long‐term remission rates of metabolic comorbidities after bariatric procedures.

Outcome	Follow‐up	LAGB	Sleeve gastrectomy	RYGB
Type 2 diabetes remission	7–15 y	20%–29% [4, 16]	26%–46% [17, 18]	33%–60% [16, 17, 20]
Hypertension remission	7–15 y	17% [4]	8%–41% [17, 18]	24%–46% [17, 20]
Dyslipidemia remission	7–15 y	23% [4]	19%–31% [17, 18]	35%–60% [17, 20]
Steatotic liver disease improvement	≥ 10 y	Limited data	Improved [19]	Reduced major adverse liver outcomes [19]
Total weight loss/excess weight loss	10–15 y	47% EWL [4]	24% TWL [18]	27%–59% EWL/TWL [16, 17, 20]

*Note:* Representative long‐term literature data are shown. Reported outcomes vary according to study population, definitions of remission, and duration of follow‐up.

Abbreviations: EWL, excess weight loss; LAGB, laparoscopic adjustable gastric banding; RYGB, Roux‐en‐Y gastric bypass; TWL, total weight loss.

Another important finding of this study was the substantial proportion of patients requiring reoperation during long‐term follow‐up. Approximately one‐quarter of patients underwent reoperation, most commonly because of device‐related complications such as band slippage and port‐related problems, or because of insufficient weight loss. These findings are consistent with previous long‐term studies reporting considerable rates of revisional surgery after LAGB [4, 5, 6]. The device‐dependent nature of LAGB therefore represents an inherent limitation of this procedure.

It should also be noted that not all band removals were associated with complications. In one patient in the present series, the gastric band was electively removed after satisfactory weight loss had been achieved at the patient's request. This observation suggests that patient preference and long‐term treatment goals may also influence the clinical course following LAGB. Although the long‐term durability of weight‐loss efficacy appears more important than reversibility in contemporary metabolic surgery practice, the potential reversibility of LAGB may still represent a unique advantage in carefully selected patients.

The present findings should be interpreted in the context of contemporary bariatric and metabolic surgery. Over the past decade, procedures with stronger metabolic effects, such as RYGB and SG, have largely replaced LAGB in clinical practice [8]. Randomized and long‐term studies have demonstrated durable metabolic improvements following these procedures [2, 3, 21]. In addition, procedures that do not rely on implanted devices are less susceptible to long‐term mechanical complications, which has contributed to the global decline in the use of LAGB reported in recent bariatric surgery registries [22]. These findings help clarify the long‐term clinical role and limitations of LAGB in the contemporary era of metabolic surgery.

Nevertheless, the present study provides valuable very long‐term outcome data from a Japanese cohort, in which such evidence remains limited. Because bariatric surgery has historically been less prevalent in Asian populations than in Western countries, long‐term observational data from Asian cohorts remain important for understanding the evolution of metabolic surgery in this region.

This study has several limitations. First, it was a retrospective single‐center study with a relatively small sample size. Second, follow‐up rates declined during long‐term observation, which may introduce bias in outcome assessment. Third, clinical management of obesity and metabolic disease evolved during the long study period and may have influenced outcomes.

In conclusion, LAGB achieved sustained long‐term weight reduction in a proportion of patients but demonstrated limited durability of metabolic improvement and a substantial need for reoperation during extended follow‐up. These findings reflect the characteristics of a purely restrictive and device‐dependent procedure. In the contemporary era of metabolic surgery, the role of LAGB appears limited and may be considered primarily in carefully selected patients with appropriate long‐term monitoring.

## Author Contributions

**Hiroomi Takayama:** conceptualization, methodology, resources, data curation, investigation. **Shun Nakamura:** resources, conceptualization. **Yuichi Endo:** conceptualization, methodology, formal analysis, writing – original draft, writing – review and editing, data curation, resources, investigation. **Hirotaka Shibata:** data curation, supervision, conceptualization, investigation. **Masahiro Kawamura:** conceptualization, resources. **Wataru Miyoshino:** conceptualization, resources. **Yoshinori Ozeki:** data curation, investigation. **Yuiko Nagasawa:** conceptualization, resources. **Masafumi Inomata:** project administration, supervision. **Yoko Kawano:** conceptualization, resources. **Takayuki Masaki:** data curation, supervision, conceptualization, investigation. **Takashi Masuda:** conceptualization, resources. **Teijiro Hirashita:** conceptualization, resources, data curation.

## Funding

The authors have nothing to report.

## Ethics Statement

All procedures performed in studies involving human participants complied with the ethical standards of the institutional and/or national research committee and with the 1964 Helsinki declaration and its later amendments or comparable ethical standards. This study has been approved by the Ethics Committee of Oita University Hospital (#3438).

## Consent

The requirement for informed consent was waived due to the retrospective nature of the study.

## Conflicts of Interest

The authors declare no conflicts of interest.

## Data Availability

The datasets used and/or analyzed during the current study are available from the corresponding author on reasonable request.
